# Prokineticin 2 (PROK2) is an important factor for angiogenesis in colorectal cancer

**DOI:** 10.18632/oncotarget.4385

**Published:** 2015-06-30

**Authors:** Hidetaka Kurebayashi, Takanori Goi, Michiaki Shimada, Noriyuki Tagai, Takayuki Naruse, Toshiyuki Nakazawa, Youhei Kimura, Yasuo Hirono, Akio Yamaguchi

**Affiliations:** ^1^ Department of Surgery, University of Fukui, Fukui 9101193, Japan

**Keywords:** colorectal cancer, prokineticin 2, angiogenesis

## Abstract

The Prokineticin 2 (PROK2) is correlated with indispensable in maintaining the homeostasis of healthy human tissues. Herein, we examined the role of PROK2 in human colorectal cancer.

After total RNA extraction from 6 colorectal cancer cell lines, we examined the expression of PROK2 mRNA. For investigating angiogenesis and tumor growth in mice, the PROK2 gene was transfected into colorectal cancer cell lines having low PROK2 mRNA expression. In addition, small interfering RNA (siRNA) was transfected into colorectal cancer cell lines having high PROK2 mRNA expression for investigation of angiogenesis and tumor growth in mice.

From 6 colorectal cancer cell lines studied, PROK2 mRNA expression was increased in 3 cell lines. When the PROK2 gene was transfected into the colorectal cancer cell line with low PROK2 mRNA expression, angiogenesis and tumor growth in mice increased significantly compared to the cell line with the control vector.

When PROK2 siRNA was transfected into colorectal cancer cell lines with high PROK2 mRNA expression, angiogenesis and tumor growth in mice were suppressed significantly compared to the cell line with siRNA (control).

This is the first report of the association of PROK2 as an angiogenic growth factor in colorectal cancer.

## INTRODUCTION

In western countries as well as in Japan, the incidence of colorectal cancer is substantially high among various malignant tumors [1–3]. Treatments for hematogenous metastasis are important for improving the survival rate of patients, because this form of metastasis has the most frequent type of recurrence [3]. Angiogenic growth factors are important factors associated with hematogenous metastasis, probably involving a number of steps of metastatic mechanisms [4, 5]. According to the currently accepted theory, an angiogenic growth factor is essential for the uptake of oxygen and nutrition by cancer cells, and new blood vessels are required especially for tumors exceeding several millimeters in diameter [6, 7]. Therefore, angiogenic growth factors are important in facilitating hematogenous metastasis. Considerable research has suggested treatment targeting vascular endothelial growth factor (VEGF) among such angiogenic growth factors to extend the prognosis of patients with unresectable colorectal cancer [8], and this treatment has been listed in NCCN's guidelines as a molecular therapy for colorectal cancer [9].

The chromosomal location of prokineticin 2 (PROK2), the gene analyzed in this study, is 3p21.1 [10]. Initially, PROK2 was stimulated in gastrointestinal peristaltic motion, but subsequent studies revealed additional roles such as promotion of steroid production, angiogenesis in the endocrine glands and heart, regulation of circadian rhythm, algesia, vascularization, and immune response [10–13]. Other recent studies found malformation of the olfactory bulb and gonadotropin-releasing hormone deficiency in PROK2/PROKR2-knockout mice and patients with Kallmann syndrome or hypogonadotropic hypogonadism harboring the PROK2/PROKR2 mutation [14–16].

This report provides insight into the mechanism of malignant colorectal tumor in association with the angiogenic growth factor PROK2.

## RESULTS

### Expression of PROK2 mRNA in colorectal cancer cell lines

**Figure 1:**
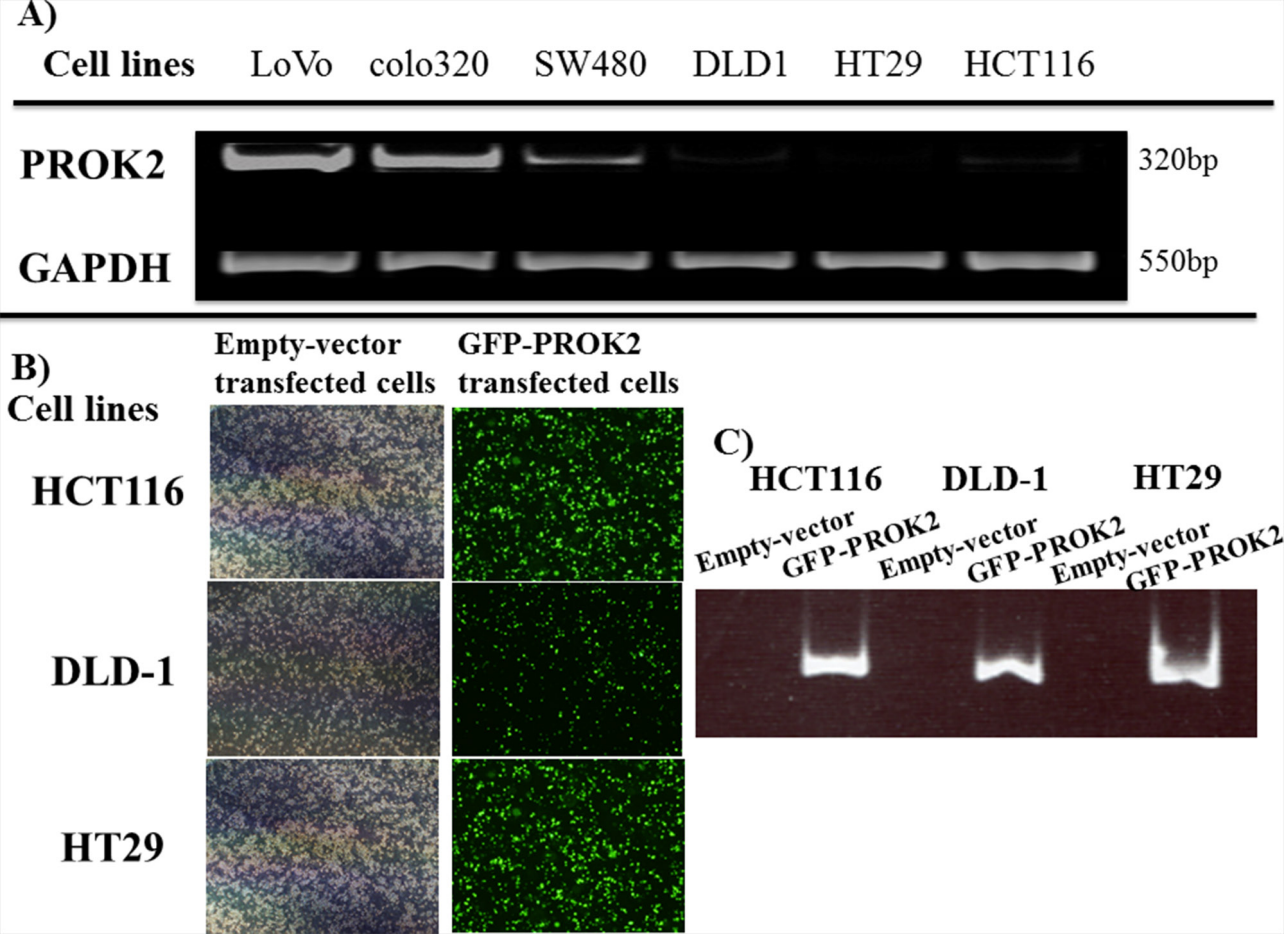
**A.** PROK2 mRNA expression in various human colorectal cancer cell lines by RT-PCR. PROK2 mRNA expression was observed in the colon cancer cell lines : LoVo, colo320, and SW480, although at different levels. **B.** PROK2 overexpressinon colorectal cancer cells(Fluorescent microscopy) Colorectal cancer cells were transfected to overexpress pcDNA3/GFP-PROK2 or pcDNA3.1-empty-vector alone. The cells expressing the appropriate protein were identified by Focal laser microscopy. Left: pcDNA3.1-empty-vector alone, Right: pcDNA3/GFP-PROK2. **C.** PROK2 overexpressinon colorectal cancer cells(RT-PCR) Expression of PROK2 mRNA was confirmed in human colon cancer cell lines transfected with pcDNA3/GFP-PROK2 or pcDNA3.1-empty-vector alone. HCT116, DLD1, HT29 cell lines; Left: pcDNA3.1-empty-vector alone, Right: pcDNA3/GFP-PROK2.

In 3 of 6 colorectal cancer cell lines, PROK2 mRNA expression occurred, and the level of expression differed across the 3 lines. PROK2 mRNA was identified by band sequencing (Figure 1A).

### Transfection of *PROK2* into colorectal cancer cell lines with low PROK2 mRNA expression

pcDNA3-GFP-PROK2 vector was transfected into colorectal cancer cell lines having low PROK2 mRNA expression (DLD-1, HCT116, and HT29). Expression(Green color) of PROK2 mRNA was confirmed by fluorescent microscopy and PROK2 mRNA (Figure 1B, 1C).

### Murine subcutaneous angiogenesis in fluid culture of colorectal cancer cell lines transfected with the PROK2 gene

**Figure 2:**
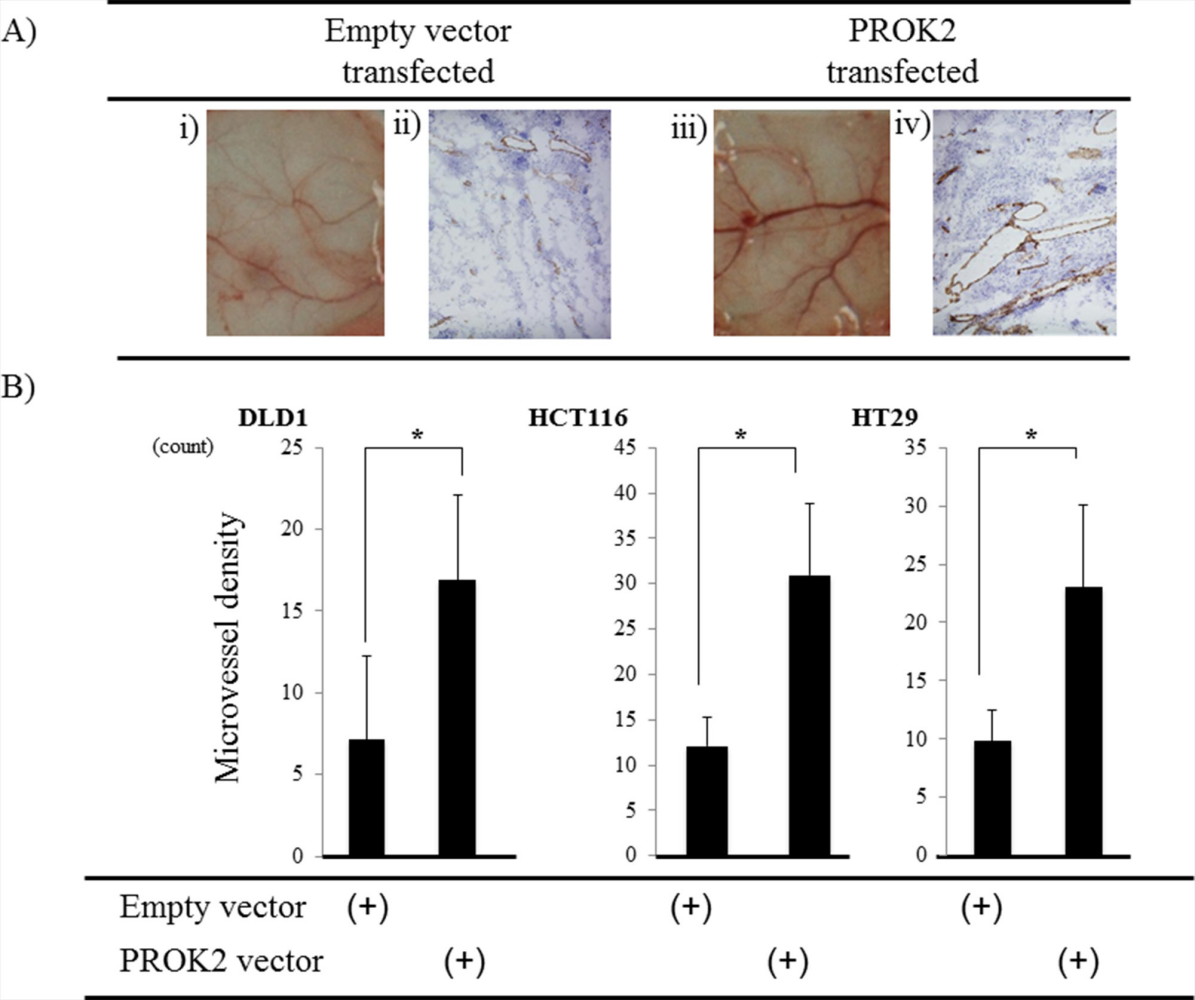
Investigation of Subcutaneous angiogenesis in mice in response to culture fluids from colorectal cancer cells transfected with the PROK2 gene **A.** i) Representative photographs of blood vessels: culture fluid alone of HCT116 cells transfected with pcDNA3.1-empty-vector. ii) CD31 expression in subcutaneous mice tissues: culture fluid alone of HCT116 cells transfected with pcDNA3.1-empty-vector. iii) Representative photographs of blood vessels: culture fluid alone of HCT116 cells transfected with pcDNA3/GFP-PROK2. iv) CD31 expression in subcutaneous mice tissues: culture fluid alone of HCT116 cells transfected with pcDNA3/GFP-PROK2. **B.** The numbers of positively CD31 stained cells in subcutaneous mice tissues. Data represent means ± SEM. (*n* = 3) (*student *t*-test *p* < 0.01)

In fluid culture of HCT116 colorectal cancer cell line transfected with the PROK2 gene vector, the size of blood vessels significantly increased compared with the cultures of colorectal cancer cell lines with the empty vector (Figure 2A-i, iii). Futhermore, immunohistochemical staining was performed using the anti-CD31 monoclonal antibody to determine the number of positively stained cells on mouse skin (Figure 2A-ii, iv). In the fluid culture with the empty vector, 7.1, 12.1, and 9.8 cells/visual field were found for DLD-1, HCT116, HT29, respectively. In the fluid culture with PROK2-containing cell lines, 16.8, 30.9, and 23.0 cells/visual field were found for DLD-1, HCT116, and HT29, respectively, showing a significant increase in the number of immunostained cells (Figure 2B).

### Tumor mass formation in mice after implantation of colorectal cancer cell lines transfected with the PROK2 gene

**Figure 3:**
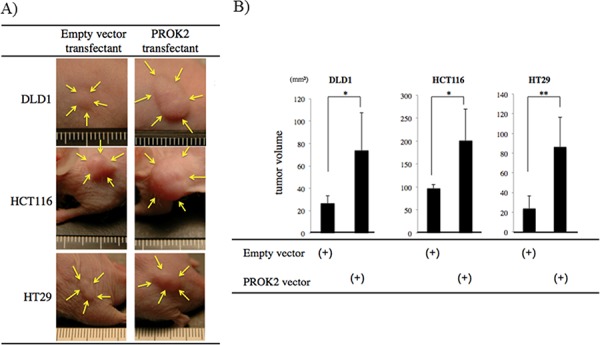
Investigation of Subcutaneous tumor formation in colorectal cancer cells transfected with the PROK2 gene Six-week-old female SHO nude mice were subcutaneously injected in the armpit region with 1.0 × 10^6^ cells colorectal cancer cells(DLD1, HCT116, HT29). Three weeks later, the tumor was resected, photographed, and weighted. **A.** Representative photographs of tumor formation Left) colorectal cancer cells transfected with pcDNA3.1-empty-vector, Right) colorectal cancer cells transfected with pcDNA3/GFP-PROK2. **B.** The measurement of subcutaneous tumor volume. Data represent means ± SEM. (*n* = 4) (*student *t*-test *p* < 0.05, **p < 0.01)

**Figure 4:**
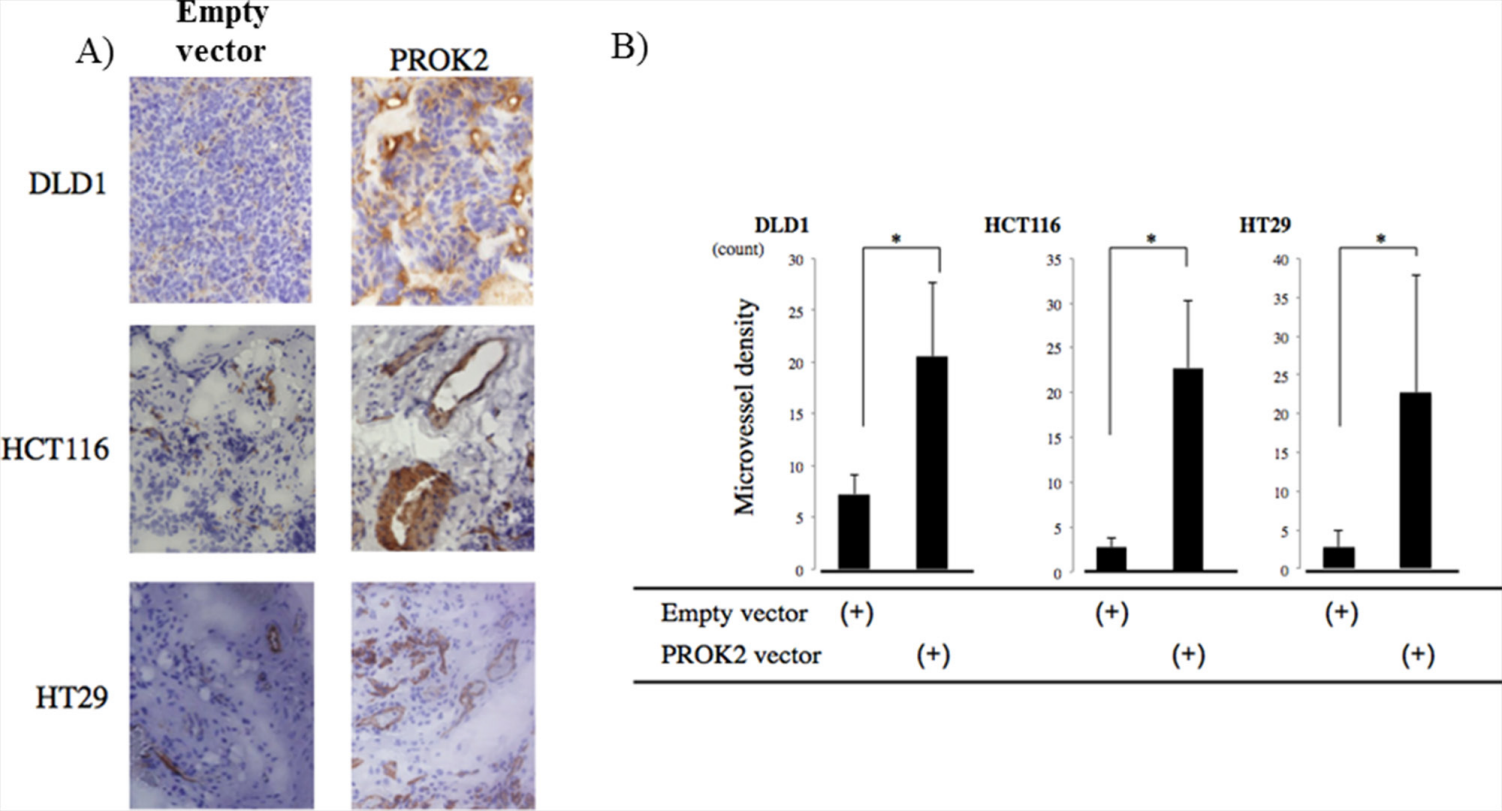
CD31 immunostaining in subcutaneous mice tumors **A.** Representative photographs Left) colorectal cancer cells transfected with pcDNA3.1-empty-vector, Right) colorectal cancer cells transfected with pcDNA3/GFP-PROK2. **B.** The numbers of positively CD31 stained cells in subcutaneous mice tumors. Data represent means ± SEM. (*n* = 4) (*student *t*-test *p* < 0.01).

After the colorectal cancer cell lines were transfected with the empty vector or the PROK2 gene vector, these cells were subcutaneously injected into mice (Figure 3A). The size of the tumor mass in 3 weeks was 26.4 mm^3^ in DLD-1, 98.0 mm^3^ in HCT116, and 23.5 mm^3^ in HT29. In contrast, after the PROK2 gene vector was transfected, the size of tumor mass was 73.6 mm^3^ in DLD-1, 201.4 mm^3^ in HCT116, and 86.3 mm^3^ in HT29, showing a significant increase in tumor mass formation (Figure 3B). Immunohistochemical staining was performed using the anti-CD31 monoclonal antibody to determine the number of positively stained cells in the tumor mass (Figure 4A). For DLD-1, HCT116, and HT29, 7.25, 2.75, and 2.44 cells/visual field, respectively, were found with the empty vector. In contrast, with the transfection of *PROK2*, the corresponding values were 20.25, 22.75, and 21.88cells/visual field respectively, showing a significant increase in the number of immunostained cells (Figure 4B).

### Suppression of murine subcutaneous angiogenesis in fluid culture of colorectal cancer cell lines transfected with Si-RNA(PROK2)

**Figure 5:**
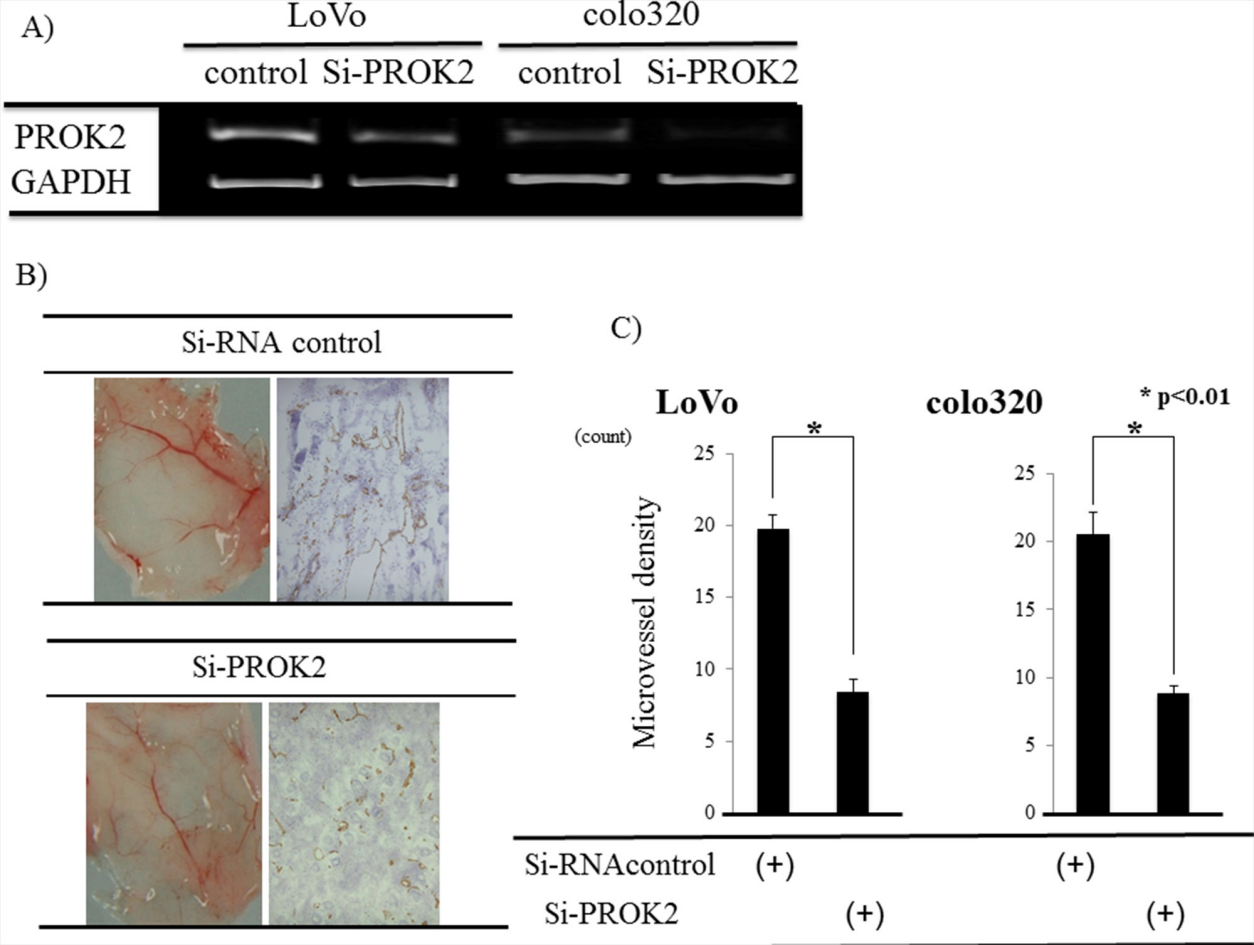
Investigation of angiogenesis in response to culture fluids from colorectal cancer cells transfected with Si-RNA(PROK2) **A.** Inhibition of PROK2 mRNA in colorectal cancer cell lines transfected with Si-RNA(PROK2) by RT-PCR. Left) LoVo cells Right) colo320 cells. **B.** Upper) LoVo cells transfected with Si-RNA(Control) Left) Representative photograph of blood vessels Right) CD31 immunostaining Lower) LoVo cells transfected with Si-RNA(PROK2) Left) Representative photograph of blood vessels Right) positively CD31 staining. **C.** The numbers of positively CD31 stained cells in the colon cancer cell lines (LoVo, colo320). Data represent means ± SEM. (*n* = 3) (*student *t*-test *p* < 0.01)

Small interfering RNA (Si-RNA; control) or Si-RNA(PROK2) was transfected into colorectal cancer cell lines with high PROK2 mRNA expression (LoVo, colo320) (Figure 5A), and angiogenic changes were examined in the fluid culture for each cell line (Figure 5B-left). Suppression of angiogenesis was macroscopically confirmed in the culture of the cell lines with Si-RNA(PROK2). Immunohistochemical staining was performed using the anti-CD31 monoclonal antibody to determine the number of positively stained cells on mouse skin (Figure 5B-right). For the cultured control cell lines, 19.8 cells/visual field were found for LoVo and 20.5 cells/visual field for colo320, while in the case of Si-RNA(PROK2)-containing cell lines, 8.4 cells/visual field were found for LoVo and 8.8 cells/visual field for colo320, showing a significant decrease in the number of immunostained cells (Figure 5C).

### Tumor mass formation in mice after injection of colorectal cancer cells transfected with Si-RNA(PROK2)

**Figure 6:**
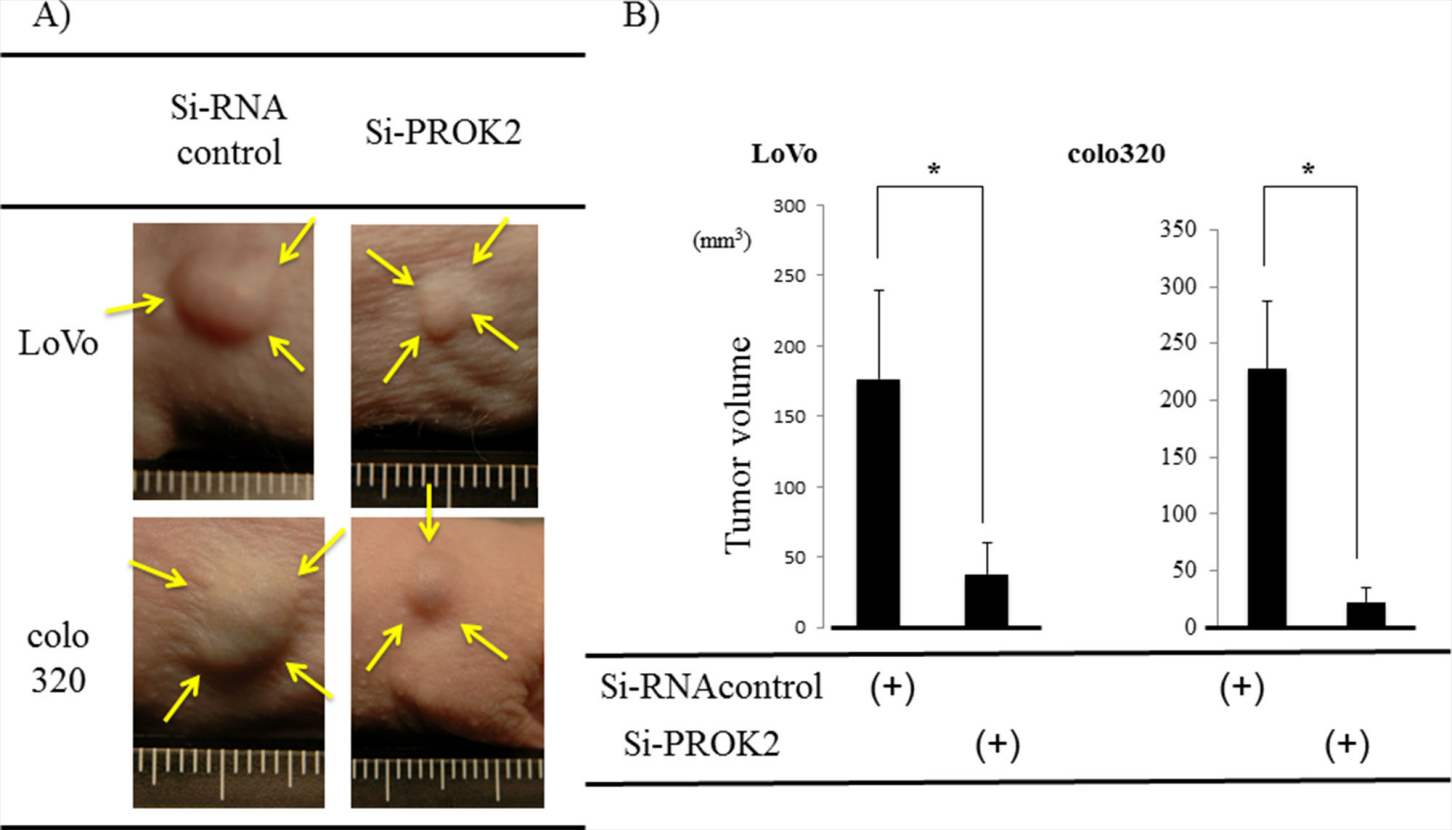
Investigation of Subcutaneous tumor formation in LoVo and colo320 colorectal cancer cells transfected with Si-RNA(PROK2) Six-week-old female SHO nude mice (Charles river, Japan) were subcutaneously injected in the right armpit region with 1.0 × 10^6^ colorectal cancer cells(LoVo, colo320). Three weeks later, the tumor was resected, photographed, and weighted. **A.** Representative photographs of tumor formation Left) colorectal cancer cells transfected with Si-RNA(Control), Right) colorectal cancer cells cells transfected with Si-RNA(PROK2). **B.** The measurement of subcutaneous tumor volume. Data represent means ± SEM. (*n* = 3) (*student *t*-test *p* < 0.01).

Si-RNA (control) or Si-RNA(PROK2) was transfected into the colorectal cancer cell lines with high PROK2 mRNA expression and subcutaneously injected in mice to examine tumor mass formation in 3 weeks (Figure 6A). The size of the mass was 176.5 mm^3^ in LoVo and 225.9 mm^3^ in colo320 when Si-RNA (control) was transfected. In contrast, the mass size was 38.1 mm^3^ in LoVo and 13.7 mm^3^ in colo320 when Si-RNA(PROK2) was transfected, showing suppression of tumor mass formation (Figure 6B).

## DISCUSSION

The metastatic mechanism of colorectal and other gastrointestinal tumors has recently been investigated from the perspective of molecular biology, and a great number of genetic alterations are activated [17–23]. Among various metastatic patterns in colorectal cancer, such as lymphogenous, peritoneal, and hematogenous metastases, hematogenous metastasis is the most frequent type of recurrence [1–3]. A clear elucidation of the actual metastatic mechanism will possibly lead to the development of novel therapies.

PROK2, which was the focus of the present study, is located at chromosome 3p21.1 and has been reported to stimulated with body functions in addition to gastrointestinal peristaltic motion, such as steroid production, promotion of angiogenesis in the endocrine glands and heart, neurogenesis, and regulation of circadian rhythm [10–13]. However, no reports have been published on the involvement of PROK2 in gastroenterological malignant tumors or its relationship with gastroenterological malignant tumors. Therefore, we decided to study colorectal cancer among various malignant tumors owing to its high incidence. First, the expression of PROK2 in colorectal cancer cell lines was examined, and the expression of PROK2 mRNA was found in many cell lines, suggesting its importance in colorectal cancer. We then focused on the relationship with angiogenesis, which is involved in hematogenous metastasis, the most critical element for post-surgery prognosis of colorectal cancer patients. We observed that vascularization and tumor growth were promoted *in vitro* and *in vivo* after PROK2 was transfected into the colorectal cancer cell lines. In terms of the structure of PROK2, it has no homology in amino acid sequence with VEGF, a well-known angiogenic growth factor, and therefore, PROK2 and VEGF are probably different proteins [10]. PROK2 might be a new angiogenic factor in colorectal cancer. Conducive environment has recently been considered the initial factor promoting the growth and development of cancer, and the role of the ambient micro-environment (niche) has been reported where angiogenic factors and various cytokines are closely related [24–28]. Considering all these findings, the colorectal cancer cell itself may promote PROK2 expression to facilitate the growth and development of cancer. The current molecular target treatments for colorectal cancer include VEGF-neutralizing agents, VEGF receptor inhibitors, EGF receptor inhibitors, and tyrosine kinase-inhibiting antibodies. The clinical use of these drugs has resulted in the improvement of the prognosis of unresectable advanced colorectal cancer patients [8, 29–32]. Nevertheless, colorectal cancer cannot be controlled completely, and the development of new therapies is necessary. We therefore examined the changes in angiogenesis and tumor growth by suppressing PROK2 expression with Si-RNA in colorectal cancer cell lines having high levels of PROK2 mRNA expression. Angiogenesis and tumor formation were significantly suppressed *in vitro* and *in vivo*, indicating that PROK2 may be developed into a new therapy in colorectal cancers with high PROK2 mRNA expression. Moreover, preliminary examination of human digestive tract tissue (colorectum and stomach) in the actual clinical setting revealed that PROK2 mRNA was not expressed in normal mucosa, while its expression was found in primary lesions of advanced digestive tract cancer and found PROK2 protein in the serum of advanced digestive tract cancer patients. Therefore, it was considered to be an important factor for metastatic mechanism in human digestive tract cancer. We have been establishing anti-PROK2 monoclonal antibody(mAb) to use for clinical trials for advanced colorectal cancer patients. In a recent preliminary study, we found PROK2 protein in the serum of advanced colorectal cancer patients.

To our knowledge, this is the first report of the association of PROK2 as an angiogenic growth factor in human colorectal cancer.

## MATERIALS AND METHODS

### Cell culture

The human colon cancer cell lines, LoVo, colo320, SW480, DLD-1, HT29, and HCT116 (obtained from European collection of cell cultures, Culture Collections of Public Health England, UK. Depositor: All cell lines were obtained from the American Type Culture Collection. ATCC) were cultured at 37C in 5% CO2 in RPMI 1640 medium(Sigma, USA) containing 10% fetal bovine serum(FBS).

### RNA extraction and reverse transcription (RT)

Total RNA was extracted from colon cancer cells using ISOGEN (Wako, Tokyo Japan). Single-strand cDNA prepared from 3 μg of total RNA using Prime Script RT reagent kit (Takara, Otsu Japan) was used as the template for the PCR [33].

### RNA extraction and Reverse transcription-polymerase chain reaction (RT-PCR) analysis

Single-strand cDNA prepared from 3 μg of total RNA using Prime Script RT reagent kit (Takara, Otsu Japan) was used as the template for the PCR. The primers for PCR to amplify Prokineticin2 (*PROK2*) (GenBank accession no. NM_021935) gene-coding regions were as follows: 5′ primer, *PROK2*-AX:5′-GGGGATCCATGAGGAGCCTGTGCTGCGCCCCA-3′; 3′primer, *PROK2*-BX:5′-GGGAATTCCTTTTGGGCTAAACAAATAAATCG-3′ [10]. Glyceraldehyde-3-phosphate dehydrogenase (GAPDH) amplification was used as internal PCR control with 5′-GGGGAGCCAAAAGGGTCATCATCT-3′ as the sense primer and 5′-GACGCCTGCTTCACCACCTTCTTG-3′ as the antisense primer. Thirty cycles of denaturation (95°C, 1 min), annealing (55°C, 1.5 min), and extension (72°C, 2.5 min) were carried out in a thermal cycler (PTC-100, Programmable Thermal Controller; MJ Research Inc., Watertown MA, USA). Ten microlitres of the PCR product were resolved by electrophoresis in 1.2% agarose gel. The sequencing was performed on PCR products that revealed the bands in RT-PCR analysis. Ethidium bromide staining of the gels identified a band of *PROK2* mRNA. To ensure reproducibility, all PCR amplifications were performed in triplicate

### Gene vector

pCMV6-PROK2 vector was obtained from OriGene company, MD, USA. pcDNA3.1-empty-vector was obtained from Invitrogen company, CA, USA. PROK2 cDNA was amplified using primers as follows: PROK2-CX, the 5′ primer, encompassed position, GGGGATCCGGTACCGAGGAGATCTGCCG, and PROK2-DX, the 3′ primer, encompassed positions, GGGAATTCGGCCGTTTAAACTCTTTCTTC. (using primers tagged with restriction enzyme sites for 5′-BamHI and 3′-EcoRI). Thirty cycles of denaturation (95°C, 1 min), annealing (50°C, 1.5 min), and extension (72°C, 2.5 min) were carried out in a thermal cycler. The BamHI and EcoRI site-tagged full length PROK2-GFP fragments were amplified and cloned into a mammalian expression vector, pcDNA3.1 (Invitrogen, CA, USA) between the BamHI and EcoRI sites. The plasmid constructs were confirmed by DNA sequencing. We established the constitutive expression vector of human PROK2-GFP (pcDNA3.1- PROK2-GFP).

### Transfection

Colorectal cancer cells were transfected to overexpress pcDNA3-GFP-PROK2 or pcDNA3.1-empty-vector alone. The colorectal cancer cells were seeded in six-well plate. Cells were transfected with appropriate amounts of plasmid DNA using Lipofectamine Plus (Invitrogen). After transfection, cells were selected for neomycin resistance by treatment with G418 sulfate (Promega, WI, USA) for 3 days. The cells expressing the appropriate protein were identified by Focal laser microscopy.

### Cell culture fluid

Each cell line was passaged at 60% confluence in a 60-mm culture plate, and cultured in RPMI1640 containing 10% FBS. The culture fluid was collected after culture of the cell lines for 3 days.

### Detection of vascularization with dorsal air sac method

As described previously [34], a Millipore chamber (Millipore; diameter, 10 mm: filter pore size, 0.45 μm) was filled with 0.2 ml culture medium of colorectal cancer cells.

The chamber was implanted into the dorsal side of six-week-old female SHO nude mice (Charles River, Japan). A rectangular incision was made in the skin on the dorsal side on Day 7 to determine the antiangiogenic effects, the chamber-contacting region was photographed.

### Small interfering RNA and transfection

Small interfering RNA (siRNA; control) or siRNA(PROK2) was were obtained from Santa Cruz Biotachnology, TX, USA. The PROK2 mRNA overexpressing colorectal cancer cell lines (LoVo, Colo320) were lipofected with siRNA transfection reagent PROK2 siRNA (Santa Cruz Biotachnology, TX, USA).

### Tumor formation in nude mice

Six-week-old female SHO nude mice (Charles river, Japan) were subcutaneously injected in the armpit region with 1.0 × 10^6^ cells in 0.1 mL of matrix gel (BD Biosciences, USA). After 21 days, the tumor was resected, photographed, and weighted. The tumor size was calculated with the formula: (L × W^2^)/2, where L is the length and W is the width of the tumor [34].

### Immunohistochemical study

Tumors and subcutaneous tisseues for histological examination were embedded in OCT compound (Sakura Finetechnical, Japan). Embedded tissues were cut into serial sections with a thickness of 4 *μ*m. Sections were gradually deparaffinized and rehydrated with xylene and ethanol. Endogenous peroxidase activity was blocked with 3% hydrogen peroxide solution for 10 minutes. Then the sections were separately incubated with anti-CD31 antibody (DAKO, Danmark) at 4°C overnight. Sections were stained by the ChemMate method using the EnVision system (DAKO). For vessel counting, one field magnified 200-fold in each of five vascularized areas was counted under microscopic observation, and average counts were recorded. Negative controls were processed with PBS instead of primary antibody.

The average of the number of microvessels in the five hotspots was recorded as the microvessel density (MVD) level of the tumor.

### Statistical analysis

Differences between two groups were analyzed by chi-square test. or Student's *t*-test using Stat Mate IV (ATMS Co., Ltd., Japan). The Cox proportional hazards model was used in multivariate regression analyses of survival date using SPSS software (IBMM SPSS Statistics, IBM Corporation, USA). Values of *P* < 0.05 were considered as statistically significant
